# Wandern während und nach Corona – Segmentierung von Wanderzielgruppen am Beispiel der Sinus-Milieus®

**DOI:** 10.1007/s00548-022-00775-5

**Published:** 2022-04-14

**Authors:** Heinz-Dieter Quack, Franziska Thiele

**Affiliations:** Karl-Scharfenberg-Fakultät Verkehr-Sport-Tourismus-Medien, Institut für Tourismus- und Regionalforschung, Ostfalia Hochschule für angewandte Wissenschaften – Hochschule Braunschweig/Wolfenbüttel, Karl-Scharfenberg-Str. 55/57, 38229 Salzgitter, Deutschland

**Keywords:** Wandertourismus, COVID-19, Zielgruppen, Überfüllung, Marktforschung, Besucherlenkung, Hiking tourism, COVID-19, Target groups, Overcrowding, Market research, Visitor management

## Abstract

Insbesondere während der Lockdowns des Jahres 2020 wurde aus einigen deutschen Regionen von überfüllten Wanderwegen und gesperrten Wanderparkplätzen berichtet, ein Zustand, der so in den betroffenen Regionen bislang noch nicht bekannt war und auf denen viele Orte und Regionen entsprechend nicht vorbereitet waren. Wer wandert eigentlich? Und kann man empirisch feststellen, dass in Deutschland pandemiebedingt mehr oder anders gewandert wurde? Sind möglicherweise „neue“ Wandernde hinzugekommen, also Personen, die präpandemisch nicht wanderten, dies aber nun für sich entdeckten und möglicherweise auch postpandemisch weiterführen? Gibt es Unterschiede innerhalb der Gruppe der Wandernden und wie kann man touristisch hinreichende Segmentierungen vornehmen? Der Beitrag reflektiert zunächst kurz tradierte Zielgruppendefinitionen, um dann detaillierter auf die möglichen vertieften Einblicke in die Genese und die Ausprägungen von Wanderverhalten einzugehen, die lebensstilorientierte Segmentierungen erlauben. Sind die „Corona-Wandernden“ andere Wandernde als die „Prä-Corona-Wandernden“ und was bedeutet dies für Wandernachfrage und Wanderverhalten ausgangs der Pandemie? Diesen Fragen widmet sich der nachfolgende Beitrag.

## Abstract (en)

Especially during the lockdowns in 2020, some regions in Germany reported overcrowded hiking trails and closed parking spaces for hikers, a condition that was previously unknown in the affected regions and for which many places and regions were not properly prepared. Who does actually hike? And can it be determined empirically that people were hiking more or differently in Germany as a result of the pandemic? Have “new” hikers possibly joined the trails, i.e. people who did not hike before the pandemic, but who have now discovered it for themselves and may continue after the pandemic? Are there differences within the group of hikers and how can one segment them for tourism purposes? The article first briefly reflects on traditional target group definitions, and then goes into more detail about the possible deeper insights into the genesis and characteristics of hiking behaviour that permit lifestyle-oriented segmentation. Are “corona hikers” different hikers from the “pre-corona` hikers” and what does this mean for hiking demand and hiking behaviour towards the end of the pandemic? These questions are addressed in the following article.

## Hintergrund

„Der neue Outdoor-Boom: Corona-Beschränkungen sorgen für Ansturm im Schwarzwald“: So titelte der SWR (2021) aktuell zu Jahresbeginn 2021. Während in den zurückliegenden Jahren insgesamt von einer tendenziell steigenden Wandernachfrage in der Gesamtbevölkerung ausgegangen werden konnte (vgl. BMWi 2010; PROJECTM GmbH 2014), scheint sich also die Wanderneigung der Bevölkerung in Deutschland pandemiebedingt nunmehr deutlich gesteigert zu haben. Sind nun die in Pandemiezeiten Wandernden die gleichen Menschen, die dies auch vor Corona getan haben, oder hat Corona dazu geführt, dass sich auch bislang Nichtwandernde dieser Outdooraktivität gegenüber geöffnet haben? Können wir also davon ausgehen, dass nun auch (zumindest vorübergehend) Menschen zu den regelmäßig Wandernden zählen, die dies bislang für sich nicht gesehen haben und unterscheiden sich diese neuen Zielgruppen von den bisherigen?

**Abb. 1:**
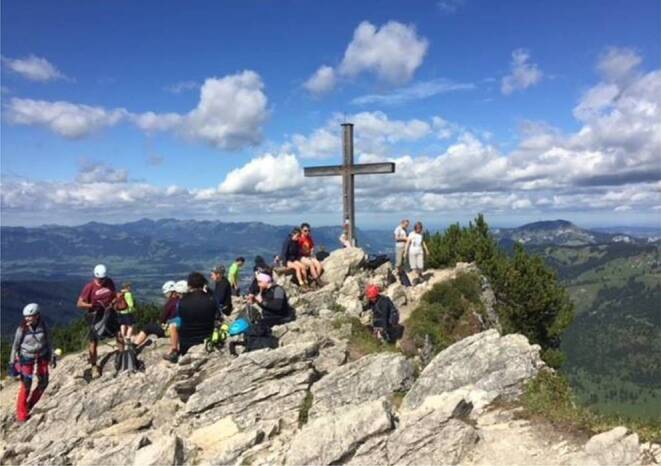
Naturschutzgebiet Allgäuer Hochalpen im Sommer 2020. (Foto: Martina Shakya)

Rund 70 % der Bevölkerung können derzeit als wanderaffin bezeichnet werden (BMWi 2010; Project M GmbH 2014; SINUS Markt- und Sozialforschung GmbH 2021a). Diese Nachfrage führt zu einem sich stetig verschärfenden Wettbewerb touristischer Zielgebiete, bei dem der Wandertourismus als wichtiger Wirtschaftsfaktor, insbesondere in ländlichen Räumen, angesehen wird (Schmude 2021). Erfolgreiche Destinationen haben eine bedarfsgerechte Infrastruktur entwickelt, die bestimmte Qualitätskriterien nach dem Deutschen Wanderverband oder dem Deutschen Wanderinstitut erfüllt. In Zeiten der Coronapandemie wird diese Infrastruktur an einigen Orten an eine Belastungsgrenze geführt und die Notwendigkeit gezielter Besucherlenkungsmaßnahmen werden deutlich (DWV 2021). Beobachtet werden konnte das auch auf stark frequentierten Berggipfeln, wie beispielsweise im Naturschutzgebiet Allgäuer Hochalpen (siehe Abb. 1).

Das liegt mitunter daran, dass insbesondere junge Menschen in den Jahren 2020 und 2021 häufiger wandern waren als vor der Pandemie (Arbeitsgruppe Wanderforschung 2021). Neue Zielgruppensegmente entdecken das Wandern für sich und es stellt sich immer häufiger die Frage, inwieweit sich Destinationen auf veränderte Nachfragestrukturen einstellen müssen. Um den Anforderungen, die Wandergäste an das touristische Angebot und die beteiligten Betriebe stellen, gerecht zu werden, sind die Destinationen auf Marktforschungsdaten angewiesen. Mit dem Wandermonitor erfasst die Arbeitsgruppe Wanderforschung der Ostfalia Hochschule für angewandte Wissenschaften alljährlich die soziodemografischen Merkmale, das Verhalten und die Anforderungen an das wandertouristische Angebot von Wandernden. Dieser Beitrag widmet sich aktuellen Veränderungen hinsichtlich der Nachfrage im Wandertourismus in Deutschland und stellt beispielhaft die Zielgruppensegmentierung nach Sinus-Milieus® vor. Die Ausführungen basieren auf den Ergebnissen des Wandermonitors (Arbeitsgruppe Wanderforschung 2019), einer im April 2021 von der Ostfalia Hochschule für angewandte Wissenschaften in Auftrag gegebenen Studie zur Wanderaktivität unter Pandemiebedingungen nach Sinus-Milieus® (SINUS Markt- und Sozialforschung GmbH, Heidelberg, Deutschland 2021a) sowie den Ergebnissen zum Reiseverhalten der Sinus-Milieus® (Juni 2021) (SINUS Markt- und Sozialforschung GmbH, Heidelberg, Deutschland 2021b).

## Wandern als Freizeit- und Urlaubsaktivität

**Abb. 2:**
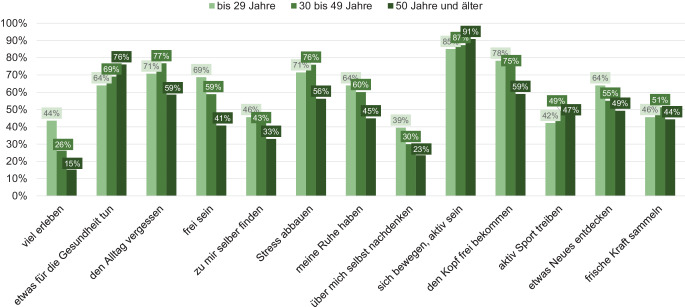
Ausgewählte Wanderanlässe nach Alter. (Quelle: Arbeitsgruppe Wanderforschung 2021, S. 21)

Der Wandertourismus ist Gegenstand verschiedener wissenschaftlicher Untersuchungen. Zu den wichtigsten Studien, die die Nachfrage umfassend charakterisiert haben, gehören die Grundlagenuntersuchung Freizeit- und Urlaubsmarkt Wandern (BMWi 2010) und die kooperativ angelegte Wanderstudie aus dem Jahr 2014 „Der deutsche Wandermarkt“ (Project M GmbH 2014). Im Vergleich dieser Studien zeigt sich eine positive Entwicklung der Gesamtnachfrage durch Abnahme des Anteils von Menschen innerhalb der Bevölkerung, die angeben nicht zu wandern und durch eine zunehmende Altersunabhängigkeit (BMWi 2010; Project M GmbH 2014). Das größte Volumen entfällt dabei auf Tageswanderungen, die 2010 mit 370 Mio. beziffert wurden (BMWI 2010). Die Arbeitsgruppe Wanderforschung führt mit dem Wandermonitor anknüpfend an die repräsentativen Wanderstudien seit 2015 jährliche Befragungen zum Wandertourismus durch. Die im Rahmen dieser Online-Befragungen im Jahr 2020 erfassten Wanderungen wurden zu 69 % in der Freizeit unternommen; 2019 waren es 64 % (Arbeitsgruppe Wanderforschung 2021). Erkennbar ist auch, dass die Wanderintensität 2020 im Vergleich zum Vorjahr angestiegen ist, was die Beobachtungen vieler Destinationen bestätigt, dass das Wandern im Zuge der COVID-19-Pandemie noch einmal an Popularität gewonnen hat. Darüber hinaus ist das Wandern eine beliebte Aktivität der Deutschen im Urlaub. Wandern ist mit 33 % der ausgeübten Aktivitäten sogar beliebter als beispielsweise das Fahrradfahren mit 22 % (FUR 2021). Dabei muss unterschieden werden in Urlaube, bei denen das Wandern das Primärmotiv der Reise darstellt und Urlaube, in denen das Wandern als eine von mehreren Aktivitäten ausgeübt wird. Im Zentrum des Interesses stehen im Urlaub genauso wie in der Freizeit das Erleben der Natur sowie die körperliche Bewegung. Hinzu kommen weitere Motive, die je nach Alter eine unterschiedlich hohe Relevanz haben und ergänzend in Abb. 2 dargestellt sind.

**Abb. 3:**
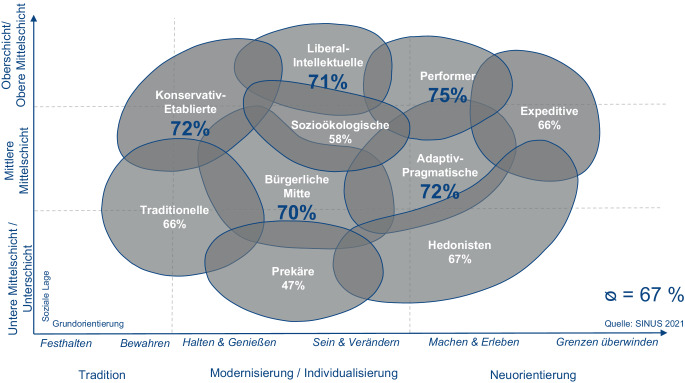
Anteile Wandernder nach Milieus (mind. selten Wandern oder häufiger) 2021. (Quelle: eigene Darstellung nach SINUS Markt- und Sozialforschung GmbH 2021a)

Das Motiv „Natur erleben“ stellt mit je rund 98 % der Nennungen über alle Befragungen und Altersgruppen hinweg das primäre Motiv einer Wanderung dar. Die Motive „sich bewegen/aktiv sein“, „etwas für die Gesundheit tun“ und „eine Region erleben“ veränderten sich in den unterschiedlichen Erhebungen kaum, sodass diese als weitere Hauptmotive des Wanderns gelten können. Ein Motivwandel wurde hingegen 2014 gegenüber 2010 in den nach innen gerichteten Motiven wie „Stress abbauen“, „frische Kraft sammeln“ und „zu sich selber finden“ und in den nach außen gerichteten Motiven, wie „neue Eindrücke gewinnen“ und „viel erleben“ festgestellt. Während die nach innen gerichteten Motive an Bedeutung gewonnen haben, hat das Interesse an Entdeckungen scheinbar deutlich abgenommen (Project M GmbH 2014). Der fortschreitende Bedeutungswandel lässt sich auch in Zeiten der Pandemie feststellen, denn die Motive „den Alltag vergessen“ und „meine Ruhe haben“ sind 2020 gegenüber 2018 in der Rangfolge wiederum um 5 bzw. 4 Plätze aufgestiegen. Möglicherweise eine Folge der mit den Einschränkungen verbundenen Herausforderungen durch Homeoffice, Homeschooling und Ausgangssperren, welche dazu führen, dass der Aufenthalt in der Natur und das Wandern einen besonderen Stellenwert eingenommen hat, während andere Freizeitaktivitäten nur eingeschränkt möglich waren. Die Motive zum Wandern sind je nach Altersklasse unterschiedlich stark ausgeprägt. In der aktuellen Zwischenauswertung des Wandermonitors des Jahres 2021 begründen die über 50-Jährigen das Wandern eher mit Gesundheits- und Bewegungsorientierung als die unter 29-Jährigen, für die Freiheit, neue Entdeckungen und die Persönlichkeitsentwicklung im Vergleich zu den Älteren beim Wandern eine höhere Bedeutung haben (Arbeitsgruppe Wanderforschung 2021). Auch was die Anforderungen an das touristische Angebot betrifft, lassen sich Unterschiede feststellen. Das Alter ist als alleiniges Abgrenzungskriterium für eine differenzierte Ansprache von Zielgruppen jedoch nicht ausreichend, denn auch andere Eigenschaften, wie z. B. die Wanderhäufigkeit oder der Kontext der Wanderung, beeinflussen die Reiseentscheidung. So werden beispielsweise Wanderungen von Menschen mit einer geringeren Wanderhäufigkeit eher im Zeitraum von April bis Oktober geplant, wohingegen Wanderungen von häufig Wandernden ganzjährig in Betracht gezogen werden (Arbeitsgruppe Wanderforschung 2021). Ob eine Person gerne wandert oder lieber Fahrrad fährt oder auch beides und wo bzw. wie häufig sie diesen Aktivitäten nachgeht, hängt also nicht allein von soziodemografischen Kriterien ab. Um wanderaffine Zielgruppen im Tourismusmarketing anzusprechen, sollte daher eine psychografische Segmentierung vorgenommen werden, bei der Persönlichkeitsmerkmale wie Einstellungen, Werthaltungen und Verhaltensweisen andere Abgrenzungskriterien ergänzen (Meffert et al. 2019). Ein Beispiel dafür ist die Segmentierung nach Sinus-Milieus®, die auf repräsentativen Marktforschungsdaten basiert. Die sozialen Milieus werden durch relativ stabile Werthaltungen in der Gesellschaft gebildet, die die jeweilige Lebenswelt charakterisieren – hier wird also nicht die tradierte Segmentierung nach soziodemografischen Merkmalen angewandt, sondern nach den die jeweiligen Personen in ihrem (Freizeit- und Konsum‑)Verhalten prägenden Wertvorstellungen (Flaig und Barth 2018; siehe auch Abb. 3). Die Ergebnisse von 2 aktuellen Studien der Sinus Markt- und Sozialforschung GmbH zeigen, inwiefern sich das Verhalten und die Anforderungen und Bedürfnisse an das Wanderangebot innerhalb der Milieus unterscheiden.

## Wandern während und nach der Coronapandemie

Eine im April 2021 durchgeführte repräsentative Online-Befragung zur Wanderaktivität nach Sinus-Milieus® in Deutschland (Fallzahl *n* = 1000) zeigt, dass im Durchschnitt 23 % der Befragten aller Milieus 2019 und 2020 regelmäßig (mehrmals pro Woche/pro Monat) gewandert sind, 25 % planen 2021 regelmäßig zu wandern. 67 % der deutschen Bevölkerung will 2021 mindestens selten wandern (SINUS Markt- und Sozialforschung GmbH 2021a). Waren es 2018 (Arbeitsgruppe Wanderforschung 2019) vor allem die traditionellen und modernen Milieus, gehen inzwischen auch neuorientierte Milieus häufiger wandern: Die Befragten aus dem Milieu der Performer und dem adaptiv-pragmatischen Milieu geben für die Jahre 2019 und 2020 eine überdurchschnittlich hohe Wanderintensität an. Die Wanderneigung innerhalb dieser Milieus ist sogar im Vergleich zu den 2018 vom Wandermonitor als wanderaffin identifizierten konservativ-etablierten und des liberal-intellektuellen Milieus angestiegen. Die Ergebnisse zeigen, dass das Wandern während der Coronapandemie auch für weitere, im Wandern bis dahin eher selten anzutreffende Milieus eine beliebte Freizeitbeschäftigung darstellt und auf einen fortschreitenden Bedeutungswandel hinweist.

Ein weiterer Studienbericht des Reiseverhaltens nach Sinus-Milieus®, der im Juni 2021 erschien (*n* = 6207 Fälle), zeigt, dass die Konservativ-etablierten und die Liberal-Intellektuellen genauso wie die Sozialökologischen aber auch die Performer während ihres Urlaubs in den letzten 3 Jahren häufig oder sehr häufig wandern waren. Auch in Hinblick auf ihre zukünftige Urlaubsplanung geben die Angehörigen des Milieus der Performer und des adaptiv-pragmatischen Milieus überdurchschnittlich häufig an, dass sie sich vorgenommen haben, häufiger wandern zu gehen als vor der Coronapandemie. Auch die Expeditiven ziehen in Zukunft häufiger einen Wanderurlaub in Betracht als andere Milieus. Im liberal-intellektuellen und dem sozialökologischen Milieu will ein größerer Anteil als in anderen Milieus genauso oft im Urlaub wandern wie zuvor, sodass diese wanderaffinen Zielgruppen darüber hinaus nicht außer Acht zu lassen sind (SINUS Markt- und Sozialforschung GmbH 2021a, b). Im Folgenden werden beispielhaft 5 der für den Wandertourismus relevanten Milieus vorgestellt.

### Das konservativ-etablierte Milieu

Die Konservativ-etablierten gehen im Urlaub gerne wandern. Die Natur ist für dieses Milieu ein wichtiges Kulturgut und heimisches Identifikationsmerkmal, um dessen Fortbestehen sich die Angehörigen sorgen (BMU 2020). Wandern im Urlaub bietet für die Konservativ-etablierten die Möglichkeit, Kraft zu tanken und Zeit mit der Familie zu verbringen, 2 der wichtigsten generellen Urlaubsmotive dieses Milieus. Im Rahmen der Reiseplanung lassen sich Angehörige dieses Milieus noch häufiger als andere persönlich beraten, sie treffen aber weniger als andere Milieus im Vorfeld ihrer Reise eine tatsächliche Buchungsentscheidung (SINUS Markt- und Sozialforschung GmbH 2021b). Im konservativ-etablierten Milieu herrscht ein hohes Traditionsbewusstsein, sodass digitale Ausrüstungsgegenstände als weniger wichtig angesehen werden als beispielsweise bequeme Wanderschuhe, Wanderstöcke, Funktionskleidung und analoges Karten- und Informationsmaterial (Arbeitsgruppe Wanderforschung 2019).

### Das liberal-intellektuelle Milieu

Auch die Liberal-intellektuellen haben einen starken Bezug zur Natur und können zumindest zur Hälfte eine attraktive Zielgruppe im Wandertourismus darstellen. Die andere Hälfte lehnt Wanderurlaube grundsätzlich ab. Auffällig ist, dass die Reisen überdurchschnittlich häufig im persönlichen Gespräch gebucht werden, auch wenn die Online-Buchung wie auch in anderen Milieus überwiegt. Abstand zum Alltag gewinnen, Spaß, Freude, Vergnügen und Natur erleben gehören zu den wichtigsten Urlaubsmotiven. Das Wandern ist ein wichtiger Ausgleich und bietet die Möglichkeit zur Entspannung sowie einen bewussten Umgang mit der Natur zu pflegen. Der Besuch von Naturattraktionen ist dabei sogar noch wichtiger als die Wanderung selbst. Dennoch ist neben dem Aufenthalt in der Natur auch die körperliche Aktivität ein wichtiger Aspekt beim Wandern, um Körper und Geist zu stärken. Die Angehörigen des Milieus informieren sich vor allem über persönliche Gespräche. Wichtig ist dabei u. a. eine fundierte und reflektierte Kommunikation (vgl. SINUS Markt- und Sozialforschung GmbH 2021b, S. 55 ff.).

### Das Milieu der Performer

**Abb. 4:**
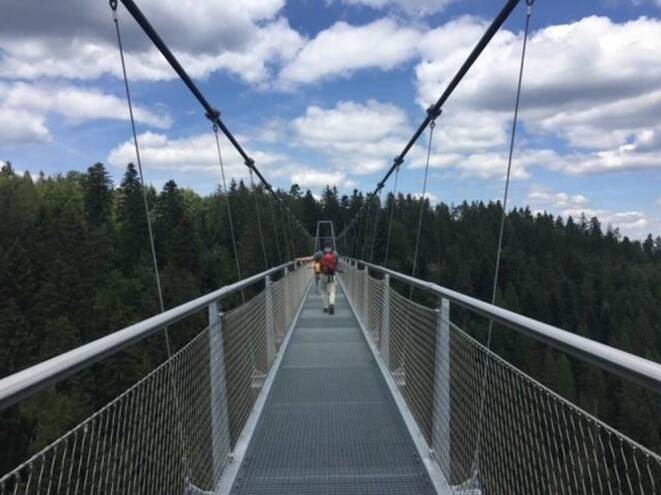
Attraktion beim Wandern: Hängebrücke Wildline, Bad Wildbad, Schwarzwald. (Foto: Martina Shakya)

Angehörige aus dem Milieu der Performer gehören wie die Konservativ-etablierten und die Liberal-intellektuellen zu den sozial gehobenen Milieus und sind 2020 überdurchschnittlich häufig gewandert. 2021 wollen sogar 38 % der Befragten aus diesem Milieu regelmäßig wandern gehen, was einer Steigerung von 46 % gegenüber 2019 entspricht. Bisher waren die Performer häufiger im Urlaub als in der Freizeit beim Wandern anzutreffen (Arbeitsgruppe Wanderforschung 2019). Die leistungsorientierten Performer unterscheiden sich zu den Konservativ-etablierten und den Liberal-intellektuellen vor allem durch ihr rationales statt emotionales Verhältnis zu Natur (vgl. BMU 2020). Bereits 2018 nutzten sie überdurchschnittlich häufig Smartphone-Apps und digitale Karten und wirkten im Vergleich deutlich multioptionaler als andere Zielgruppen, z. B. bei der Wahl der Wege oder der Freizeitaktivitäten (Arbeitsgruppe Wanderforschung 2019). 50 % der Angehörigen dieses Milieus geben an, in den letzten 3 Jahren im Urlaub häufig oder sehr häufig gewandert zu sein. Das Wandern im Urlaub dient in diesem Milieu vor allem auch der Fitness und wird ausgeübt, um Kraft für den Alltag zu schöpfen. Bei der Reiseentscheidung hat die Nähe zum Wohnort, Aus- und Weitblicke (siehe auch Abb. 4), Kinderfreundlichkeit der Wege und persönliche Weiterentwicklung eine höhere Bedeutung als in anderen Milieus (SINUS Markt- und Sozialforschung GmbH 2021a, b).

### Das sozialökologische Milieu

Das sozialökologische Milieu ist stärker im Urlaubs- als im Freizeittourismus zu finden. Im Vergleich zu anderen Milieus präferieren die Angehörigen dieses Milieus bei längeren Urlauben eher Ferienwohnungen und -häuser als Hotels. Bei Kurzurlauben hat die Bahn als Reiseverkehrsmittel eine überdurchschnittlich hohe Bedeutung. Grundsätzlich prägt das Thema Nachhaltigkeit den Lebensstil der Sozialökologischen, sodass die Vielfalt und der Schutz der Natur einen besonders hohen Stellenwert haben (BMU 2020). Beim Reiseverhalten äußert sich das darin, dass die Natur zu erleben, neben dem Abstand zum Alltag, Entspannung und dem Auftanken eines der wichtigsten Urlaubsmotive ist. Dabei ist beim Wandern weniger die körperliche Aktivität wichtig als vielmehr die Stärkung des Wohlbefindens durch die bewusste Aneignung der Natur, die als Kraftquelle zum mentalen Auftanken angesehen werden kann. Wie auch bei den anderen Milieus dienen Freunde, Verwandte und Bekannte als wichtige Informationsquellen bei der Reiseentscheidung (SINUS Markt- und Sozialforschung GmbH 2021b).

### Das adaptiv-pragmatische Milieu

Die Bedeutung des Wanderns im Milieu der Adaptiv-pragmatischen hat in den vergangenen 3 Jahren in der Freizeit zugenommen, Wandern im Urlaub spielt dagegen kaum eine Rolle. Bei den Wanderungen steht das persönliche Wohlbefinden im Fokus. Es sind vor allem die Motive „etwas für die Gesundheit tun“, „frische Kraft sammeln“, „frei sein“, „zu mir selber finden“, „über mich selbst nachdenken“ und „in Geselligkeit sein“ relevanter als in anderen Milieus. 2020 wurde neben der Möglichkeit an der frischen Luft bzw. draußen zu sein, die Natur zu erleben und in Bewegung zu sein, das Wandern in dieser Zielgruppe überdurchschnittlich häufig als Urlaubsersatz angesehen. Es ist davon auszugehen, dass es schwierig sein wird, diese Zielgruppe im Wandertourismus langfristig zu binden. Wanderangebote oder das Erleben der Natur werden nicht gezielt gesucht. Stattdessen stehen auf Reisen vor allem Spaß, Entspannung und Abwechslung im Fokus. Leichte sportliche Aktivitäten und Ausflüge in die Umgebung nehmen als Aktivitäten im Urlaub einen größeren Stellenwert ein. Angehörige dieses Milieus nutzen Wanderwege aufgrund besonderer Attraktionen, wie z. B. Hängebrücken (siehe Abb. 4). Durch ihre Multioptionaliät kommen sie daher eher spontan und in Kombination mit anderen Aktivitäten zum Wandern (SINUS Markt- und Sozialforschung GmbH 2021a, b).

## Fazit und Ausblick

Die vorgestellten Ergebnisse verdeutlichen, dass sich der fortschreitende Bedeutungswandel im Wandertourismus in veränderten Nachfragestrukturen manifestieren kann. Neben der Natur und der Bewegung haben je nach Milieu einzelne Aspekte beim Wandern eine höhere Bedeutung als andere und damit einen Einfluss auf den Reiseentscheidungsprozess und die Beurteilung des Urlaubserlebens in den Destinationen. Natur und Landschaft stellen im Wandertourismus einen individuellen Erlebensraum dar, der anhand von Wanderwegen erschlossen wird. Sowohl bei der Kommunikation als auch bei der Erschließung dieses Erlebensraumes hilft die differenzierte Betrachtung der Milieus, um die unterschiedlichen Motivationen und Erwartungen dabei entsprechend zu berücksichtigen und die jeweiligen Zielgruppen unterschiedlich zu adressieren. Je nach Milieu haben der Aufenthalt in der Natur bzw. naturnahen Umgebung und die körperliche Aktivität einen unterschiedlichen Stellenwert. Das Wandern selbst ist dabei immer häufiger Mittel zum Zweck als die zentrale Hauptmotivation eines Urlaubs. Pandemiebedingt haben sich nicht nur mehr Menschen in der Freizeit zu Fuß auf den Weg gemacht, sondern auch andere als die bis einschließlich 2019 bekannten Wandernden: Die bislang vorliegenden Daten zeigen, dass gerade etwas jüngere Menschen, die bislang in der Marktforschung als eher wanderfern eingestuft wurden, ergänzend zum klassischen Wanderpublikum dieser Aktivität nachgingen. Diese modernen und neu wanderorientierten Zielgruppen weisen ein geringeres Naturbewusstsein auf, sodass erwartet werden kann, dass sie sich unerfahrener und unsensibler in der Landschaft bewegen. Durch den Einsatz digitaler Medien können sie jedoch auch für Wege und Regionen gewonnen werden, die bisher weniger ausgelastet waren. Einerseits ergeben sich so neue Möglichkeiten der Besucherlenkung und Wegenutzung in der Fläche, andererseits können sich bestimmte Orte durch eine schnelle Verbreitung über Social-Media-Kanäle auch rasch zu Hotspots entwickeln, deren Belastungssituation dann kaum noch zu steuern ist. Inwiefern sich die Erhöhung des Besucheraufkommens und eine solche räumliche Umverteilung in den Destinationen bemerkbar machen, muss deutschlandweit genauer untersucht werden. Insbesondere ist derzeit noch unklar, ob die gestiegene und zu einem deutlichen Teil offenbar pandemiebedingte Wanderorientierung von Teilen der Bevölkerung auch postpandemisch bleibt oder nach einer Übergangszeit wieder auf das Vorkrisenniveau sinkt.
